# 2114: The role of estrogen receptor-β in the development of the early endometriotic lesion

**DOI:** 10.1017/cts.2017.203

**Published:** 2018-05-10

**Authors:** Jennifer Knudtson, Ya-Guang Liu, Marlen Tellez Santos, Rajeshwar Tekmal, Ratna Vadlamudi, Robert Schenken

## Abstract

OBJECTIVES/SPECIFIC AIMS: To further elucidate the role of estrogen receptor β (ER-β) in the early endometriotic lesion attachment. METHODS/STUDY POPULATION: EECs were immortalized using a telomerase vector. Immortalized cells and parental cells were characterized by genotyping, and expression of ER-β as well as other epithelial cell markers. ER-β was knocked-down in immortalized EECs using lentivirus-mediated shRNA transduction. ER-β knockdown was confirmed by RT-qPCR and Western analysis. EEC cells with or without ER-β knockdown were used to assess their attachment to PMCs in an established in vitro assay (Lucidi, 2005). Results were analyzed with Student *t*-test. RESULTS/ANTICIPATED RESULTS: Genotyping using karyotype assay confirmed a normal chromosomal profile. Also positive staining for cytokeratin and lack of any staining with vimentin confirms the epithelial origin of these cells. ER-β knockdown has a significant decrease in attachment compared to control (*p*=0.02). DISCUSSION/SIGNIFICANCE OF IMPACT: Primary and immortalized cells were 46XX, cytokeratin positive, and vimentin negative confirming their epithelial origin. ER-β knockdown has a significant decrease in attachment compared with control.

